# Binary grading may be more appropriate for endometrial cancer

**DOI:** 10.4274/jtgga.galenos.2019.2019.0068

**Published:** 2020-09-03

**Authors:** Kazibe Koyuncu, Duygu Altın, Batuhan Turgay, Bulut Varlı, Bahar Konuralp, Yavuz Emre Şükür, Salih Taşkın, Fırat Ortaç

**Affiliations:** 1 Department of Obstetrics and Gynecology, Ankara University Faculty of Medicine, Ankara, Turkey; 2 Clinic of Obstetrics and Gynecology, University of Health Sciences Turkey, Tepecik Traning and Research Hospital, İzmir, Turkey

**Keywords:** Endometrial cancer, prognostic factor, survival, peritoneal cytology, grade

## Abstract

**Objective:**

To elucidate the survival consequences of the prognostic factors for endometrial cancer.

**Material and Methods:**

This was a retrospective study of 276 patients diagnosed with endometrial cancer who admitted for staging surgery. The extent of the surgery was determined by clinical staging and preoperative evaluation. The pathology specimens were reassessed by a gynecopathologist. Independent risk factors were revealed for the progression-free survival (PFS), overall survival (OS) and disease-specific survival (DSS) utilizing Kaplan-Meier and “Cox” proportional analysis.

**Results:**

The median follow up of the patients was 50 months. Of the 29 patients who died, 15 (5.43%) died because of endometrial cancer. Multivariate analysis revealed that independent risk factors for OS and PFS were stage (p=0.002, 0.002, respectively) and grade 3 (G3) histology (p=0.013, 0.015, respectively). Positive peritoneal cytology was an independent risk factor for OS (p=0.024), but not for PFS (p=0.050). Stage (p=0.005) was found to be the only independent risk factor for DSS. Patients with G1 and G2 histology had a similar and more favorable prognosis than patients with G3 histology.

**Conclusion:**

Advanced stage, high-grade tumor and the presence of positive peritoneal cytology were ascertained as independent prognostic factors for endometrial cancer. A binary histological grading system could be simpler and as effective as the current three grade system because grade 1 and 2 patients showed similar prognosis.

## Introduction

Endometrial cancer is the most commonly diagnosed gynecologic malignancy in the USA and European countries (1). Two different subtypes of endometrial cancer have been defined as their pathogenesis and outcomes differ. Approximately 80% of the patients are diagnosed with type 1 (endometrioid) cancers which are estrogen related, more than 70% of the cases have stage 1 disease at diagnosis, and the five-year overall survival is approximately 83%. Type 2 non-endometrioid cancers are seen in elderly women, recognised at more advanced stages, and outcomes are worse (2,3).

The leading prognostic factor for endometrial cancer survival rates is the stage. Stage 1 patients have 91% overall survival (OS) whereas stage 4 patients have rates of 30% (4). In addition to the stage, many other prognostic factors play an important role for survival, such as age, histologic subtype, lymphovascular space invasion (LVSI), myometrial invasion (MI), histologic grade, and tumor size (4,5,6). Positive peritoneal cytology was removed from the International Federation of Gynecology and Obstetrics (FIGO) staging system. However, it should be noted that its prognostic significance is still controversial (7,8,9,10,11,12). In recent years, the presence of LVSI has gained importance (6,13,14). Risk of recurrence and treatment is stratified, based on these prognostic factors (15,16,17).

This study was carried out to elicit the effects of prognostic factors on different types of survival, such as OS, progression-free survival (PFS) and disease-specific survival (DSS) in patients who were treated with staging surgery for endometrial cancer at a single tertiary institutional center.

## Material and Methods

### Patients

A retrospective observational study was conducted in a tertiary center. Institutional review board approved the study (IRB approval number: 10-42014, date: June 9th, 2014). Data of 303 patients diagnosed with endometrial cancer and treated between January 2005 and February 2014 at the Department of Gynecologic Oncology were reviewed. Patients with previous or concurrent primary cancers, who were not treated surgically, whose follow-up information was missing and surveillance time less than six months were excluded from this study. Eventually, 276 patients were selected for the study. The pathology specimens of all patients were re-evaluated by an expert gynecopathologist who has worked in this field for more than 30 years to ensure the accuracy of the diagnosis.

Surgical treatment consisted of total abdominal hysterectomy and bilateral salpingo-oophorectomy or bilateral salpingectomy, including pelvic/paraaortic lymph node dissection according to the circumstance, omentectomy and peritoneal cytology assessment. Adjuvant treatment after surgery was decided after each patient was presented and discussed at the tumor board in the light of the guidelines. Adjuvant treatment was administered as radiotherapy (RT) and/or chemotherapy (CT), including cisplatin and/or doxorubicin according to tumor characteristics and practices at the time. External beam RT and/or intravaginal brachytherapy (BT) was administered to the patients for RT. Adjuvant treatments of the patients were given in the same tertiary center, in the medical oncology or radiation oncology centers.

Demographic information, clinicopathologic features and survival status for women diagnosed with endometrial cancer and fulfilling the inclusion criteria were collected from the hospital medical records (95% of the data), national database (1% of the data) and via telephone calls with patients (4% of the data). Data included age, menopausal status, parity, extent of surgery, stage, number of dissected lymph nodes, histological subtype, tumor size, tumor grade, MI, LVSI, cervical tissue involvement, peritoneal cytology, the kind of adjuvant therapy, the appointment date and status of the patient at latest follow-up, date and location of recurrence and time of death, if applicable. Patient’s co-morbidities were also documented: 75 (26.4%) of the patients had diabetes mellitus; 125 (45.2%) of them had hypertension; 32 (11.5%) had cardio-vascular disease; and 11 (0.03%) had other malignancy (four breast cancer, four colon cancer, one lung cancer, one multiple myeloma and one osteosarcoma). In order to avoid bias, disease specific and OS rates were calculated separately. For the histological classification and grading, World Health Organization criteria were used (18). Peritoneal cytology samples were obtained by either taking the fluid which is already present in the intra-abdominal cavity or after splashing the intra-abdominal cavity with 100 mL saline. The existence of malignant cells, regardless of the number, was considered to be positive peritoneal cytology.

Patients proceeded to follow up protocol after the treatment, which was every three months after the surgery for the first two years, every six months for the next consecutive three years, and subsequent annual visits were suggested.

OS was described as lifetime between initial surgery to death from any kind of reason, DSS as the lifetime between first surgery to death from disease and PFS as for the time from the initial surgery to the initial recurrence. If patient did not have recurrence or had died then OS, DSS and PFS were determined as the length of time from surgery until the last follow-up visit.

Informed consent was taken from all patients in this study.

### Statistical analysis

The normality of distribution was tested by Shapiro-Wilk test. According to the results nonparametric tests were preffered. Continuous data are peresented as median (min-max). Categorical data are presented as frequency (percentage).

Univariate and multivariate analysis were used to determine independent prognostic factors. Cox proportional hazard regression analysis was used to determine hazard ratio (HR) for survivals and the ratio of increased hazard for recurrence and death.

The Kaplan-Meier method was used to establish survival curves for OS, DSS and PFS. The differences between groups were compared using the Log-rank test. Statistical analyses were performed using SPSS software, version 15.0, (IBM Inc., Chicago, IL, USA) and a p-value of less than 0.05 was noted to be statistically significant.

## Results

Median (range) age of the 276 patients was 60 (25-86) years. The median follow-up time was 50 (6-141) months. Table 1 shows the demographic and clinical features. Two hundred sixty-four patients (95.7%) had endometrial cancer with endometrioid histology. Twelve patients (4.3%) had non-endometrioid histology, which consisted of seven serous, two mucinous, one clear cell, one mixed and one neuroendocrine tumor.

Surgery included total hysterectomy and bilateral salpingectomy or salpingo-oophorectomy in all cases. In 244 cases (88.4%), pelvic lymphadenectomy was performed, with a median of 26 (3-76) lymph nodes removed. One hundred and twenty-four cases (44.9%) of the patients underwent paraaortic lymphadenectomy with a median of 9 (1-36) lymph nodes removed. Peritoneal washings were obtained from 208 patients (75.4%). Nine patients (4.3%) had positive cytology.

One hundred thirty one patients (47.5%) received adjuvant therapy. Eighteen patients (6.5%) received CT alone, 89 (32.2%) received radiation therapy alone, and chemo-radiation was administered to 24 patients (8.7%). Of the 113 patients who received RT, 68 received BT alone, 17 received external beam radiation therapy alone. Of the remainder, four patients were administered extended field radiation therapy and 24 were administered combined BT and external beam radiation therapy.

A total of 29 patients died, of whom 15 (5.43%) died due to the endometrial cancer. Stage, grade, histologic subtype, LVSI, age, having positive peritoneal cytology and the administration of adjuvant therapy were risk factors regarding OS in univariate analysis. Among the variables stage, grade and positive peritoneal cytology were shown to be independent risk factors in multivariate analysis. Factors evaluated for an association with OS are summarized in Table 2.

**Figure 1:**
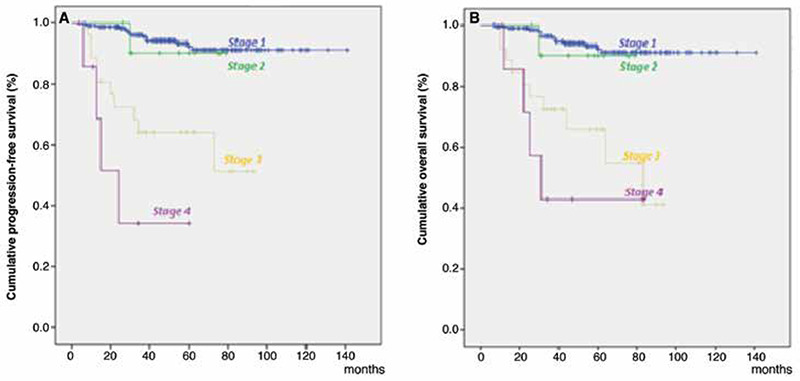
Kaplan-Meier survival curves for the stage. A) Progression-free survival stratified by stage. B) Overall survival stratified by stage

**Figure 2:**
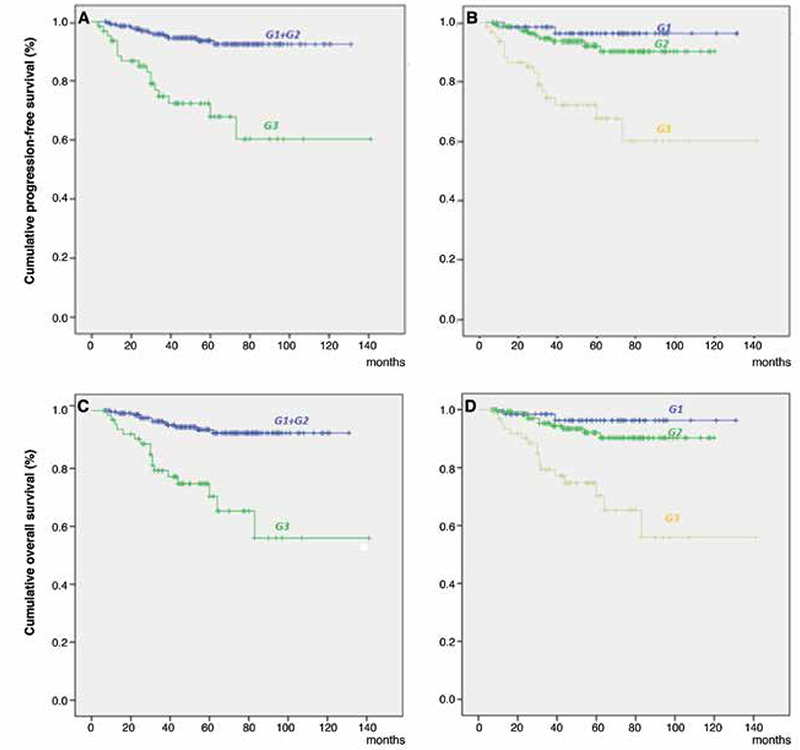
Kaplan-Meier survival curves for prognostic factors. A) Disease-specific survival stratified by two-tiered grade. B) Disease-specific survival stratified by three-tiered grade. C) Overall survival stratified by two-tiered grade. D) Overall survival stratified by three-tiered grade

The 5-year OS was 92.1% for patients with stage 1 disease, 90% for patients with stage 2 disease, 65.9% for patients with stage 3 disease and 42.9% for patients with stage 4 disease. Figure 1 presents the Kaplan-Meier survival curves of patients based on stages (p=0.002). The 5-year OS was 96.3% for G1 disease, 92.1% for G2 disease and 70.3% for G3 disease. When the patients were recategorized as G1+2 and G3, 5-year OS was found to be 93.4% for G1+2 disease and remained 70.3% for G3 disease. Figure 2 shows the Kaplan-Meier curves of patients according to three- and two-tiered FIGO grades respectively.

**Figure 3:**
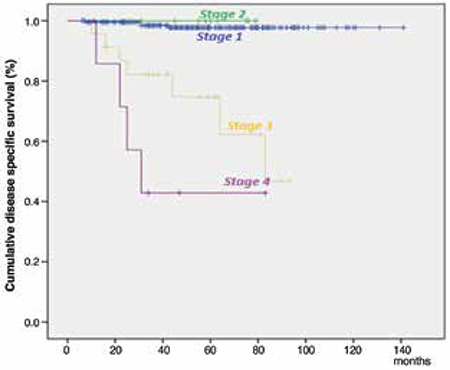
Kaplan-Meier survival curves for stage

The 5-year DSS was 97.7% for stage 1, 100% for stage 2, 74.7% for stage 3 and 42.9% for stage 4 (Figure 3). While stage, grade, histologic subtype, LVSI, MI, positive peritoneal cytology and the administration of adjuvant therapy were risk factors for DSS on univariate analysis, stage remained the only independent variable (p=0.005) associated with poor DSS in multivariate analysis (Table 2).

Thirty patients (10.9%) developed recurrences. Recurrences occurred at a median (range) time of 23 (3-86) months. Distribution of the recurrences’ regions were as follows: vaginal apex (n=4), pelvis (n=2), lymph nodes (n=8), abdominal (n=4) and distant (n=4). The remaining nine patients had recurrences in two different areas. Recurrences were seen in 24 (9.1%) of the 264 patients with endometrioid histology and 6 (50%) of the 12 with non-endometrioid histology.

The 5-year PFS was 92.2% for stage 1 EC, 90% for stage 2 EC, 63.9% for stage 3 EC and 34.3% for stage 4 EC (p=0.002) (Figure 1). Stage, grade, histologic subtype, LVSI, age, administration of adjuvant therapy and having positive peritoneal cytology were shown to be significantly related with PFS in univariate analysis. Stage and grade retained independent significance in the multivariate analysis (Table 2). There was no statistically significant difference between the outcomes of grade 1 and 2 patients (p=0.475). The 5-year PFS of patients with grade 1, 2 and 3 tumors was 96.3%, 92.3% and 67.8% respectively (p=0.015) (Figure 2). When grade was recategorized as a binary system, the 5-year PFS was found to be 93.5% for G1+2 disease and remained 67.8% for G3 disease (p=0.015) (Figure 2).

## Discussion

Grade has been shown to be an important prognostic factor in many studies (19,20). Consistent with the literature, the grade was found to be an independent factor regarding survival in our study. However, the current FIGO grading system’s reproducibility, ease of use and prognostication are being debated (21). Some studies showed that grade 1 and 2 tumors had similar survival rates which were better than grade 3 tumors (22,23,24). Consistent with this, our results showed that both OS and PFS rates were not statistically different regarding G1 and 2 tumors and better than G3 tumors (p=0.015 and p=0.015, respectively).

Furthermore, grade 2 tumors are not consistent in defining the recurrence risk and necessity for postoperative adjuvant treatment. Colombo et al. (15) described a guideline for “European Society for Medical Oncology” defining prognosis, treatment and follow-up of endometrial cancer which emphasized that the decision for giving adjuvant therapy does not differ between G1 and G2 patients for stage 1A and B. Studies showed that G3 endometrioid tumors do not differ compared to patients who have papillary serous or clear-cell histology and should be considered and treated as type 2 endometrial cancers (25,26). According to early evidence and our study, it may be more practical and efficient to use a simple binary grading system where G1 and 2 were classified as a single group.

Stage is accepted as the best prognostic factor to predict survival in endometrial cancer as advancing stage is related to a poorer OS and PFS (27). The approximate 5-year survival rates for stage 1, 2, 3 and 4 EC disease are 80-90%, 80%, 50-70% and 20% respectively (3,4). Except for higher survival rates of stage 2 patients with EC and stage 4 patients with EC, survival rates in our study are in accordance with the literature, as there was a significant reduction with advancing stages. An explanation for this finding may be related to the relatively small number of patients with stage 2 (n=11) and 4 (n=6) disease in our population.

Given that peritoneal cytology is not a part of surgical staging, the relationship between survival and peritoneal cytology particularly is still very controversial in early-stage patients. While some studies showed positive peritoneal cytology is related to high rates of recurrences and poor survival (7,10,11), some found out no relationship between positive peritoneal cytology and survival (28,29). Seagle et al. (30) analyzed data from the National Cancer Database and reported that adjuvant CT provides better survival in patients who were diagnosed as an early endometrioid type of endometrial cancer patients with positive peritoneal cytology. In the literature, it was suggested that having positive peritoneal cytology might be related to worse prognosis in alliance with other adverse prognostic factors (31). In the literature non-endometrioid histology was shown to be related to positive peritoneal cytology (31). In the present study there were only 12 non-endometrioid patients, and thus it was not possible to draw any firm conclusion, due to the low number of patients. Furthermore, in our cohort positive peritoneal cytology incidence was 4.3% and less than in the literature (8,32). This may be related to the low number of high-risk patients. Although positive peritoneal cytology emerged as an independent prognostic factor for OS (p=0.024, HR: 5.8, 95% confidence interval: 4.98-7.01), it did not quite achieve statistical significance in the multivariate analysis (p=0.050) but was also related with poor PFS (p=0.009). Despite not conducting a subgroup analysis, our findings support the suggestion that positive peritoneal cytology adversely affects survival besides grade, irrespective of the given adjuvant therapy.

MI has long been recognized as a prognostic factor (19). Although it did not reach statistical significance, MI deeper than half of the myometrium was associated with shorter OS and PFS (p=0.057, and p=0.05, respectively). For DSS, MI was shown to be a poor prognostic factor but not an independent one (p=0.026). The decrease in the survival rates could be explained by increased lymph node involvement with deeper MI. In previous studies, deep MI was reported to be related to higher rates of nodal involvement (19,33).

LVSI is another well-documented prognostic factor in endometrial cancer. Patients with LVSI have 5.8 times the increased risk of recurrence (34). Guntupalli et al. (13) showed that LVSI had a 95% negative predictive value for nodal disease. In the present study, we also showed that patients with LVSI have poorer survival rates compared to the patients without LVSI. In addition to this, being a prognostic factor, LVSI is also used in the risk stratification systems. Several authors place LVSI positive patients into the high-intermediate risk category and suggest these patients could benefit from adjuvant RT (35,36,37).

A strength of this study is the uniform management of patients since this study was conducted in a single center. Surgical management of the patients differs between countries and even within countries. In our clinic, full staging with pelvic and para-aortic lymphadenectomy in the 2000s and early 2010s was preferred, which comprised most of the patients in this study and enabled us to know the definite stage of the patients and administration of the adjuvant therapy accurately. After abandoning lymphadenectomy in low-risk patients, some of the advanced staged patients stay under-staged which is another issue that is an ongoing debate. Uterine risk factors and adjuvant treatments are known in detail in this study. Another strength of the study is that patients were monitored for a long time and this has enabled us to better understand risk factors for recurrence.

### Study limitations

The limitations of the study are the retrospective design of this research, small numbers of patients in two of the EC grading groups and the small number of patients with positive cytology.

## Conclusion

Advanced stage, grade 3 tumor and positive peritoneal cytology were regarded as independent prognostic factors for endometrial cancer. Since grade 1 and 2 tumors show similar prognosis, a binary grading system combining these two grades could be simpler. Removing grade 2 from the current grading system may also improve risk stratification and help to eliminate confusion regarding adjuvant therapy.

Positive peritoneal cytology is not a part of staging, but in several studies, including the present study, the findings showed that positive peritoneal cytology is a poor prognostic factor. Thus, it may be clinically useful for risk stratification to plan adjuvant treatment.

## Figures and Tables

**Table 1:**
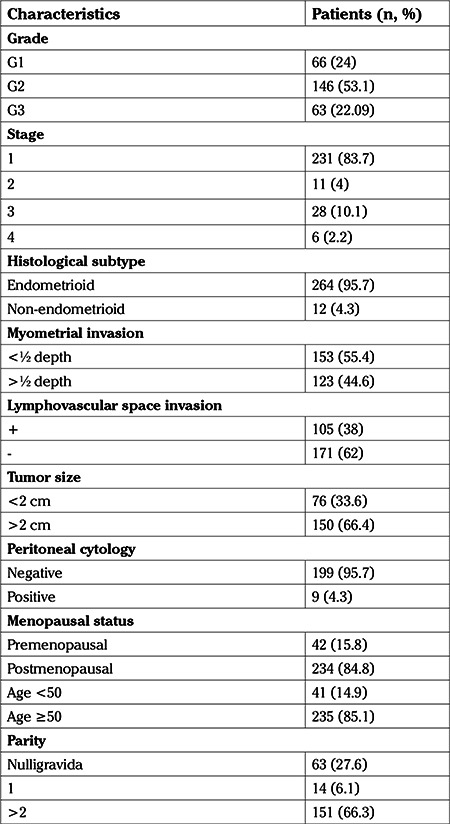
Characteristics of the endometrial tumors in two hundred seventy-six patients

**Table 2:**
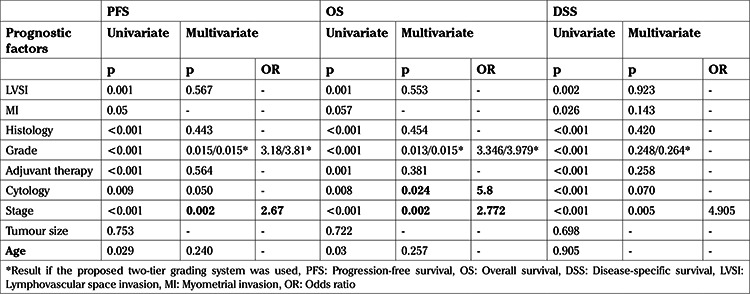
Multivariate and univariate analyses of the prognostic factors
